# Effects of a discoloration-resistant calcium aluminosilicate cement on the viability and proliferation of undifferentiated human dental pulp stem cells

**DOI:** 10.1038/srep17177

**Published:** 2015-11-30

**Authors:** Li-na Niu, Devon Watson, Kyle Thames, Carolyn M. Primus, Brian E. Bergeron, Kai Jiao, Eduardo A. Bortoluzzi, Christopher W. Cutler, Ji-hua Chen, David H. Pashley, Franklin R. Tay

**Affiliations:** 1 State Key Laboratory of Military Stomatology, School of Stomatology, Department of Prosthodontics, Fourth Military Medical University, Xi’an, Shaanxi, China; 2 Department of Endodontics, Georgia Regents University, Augusta, Georgia, USA; 3 LECOM School of Dental Medicine, Bradenton, Florida, USA; 4 Department of Dentistry, Federal University of Santa Catarina, Florianópolis, Santa Catarina, Brazil; 5 Department of Periodontics, Georgia Regents University, Augusta, Georgia, USA; 6 Department of Oral Biology, Georgia Regents University, Augusta, Georgia, USA

## Abstract

Discoloration-resistant calcium aluminosilicate cement has been formulated to overcome the timely problem of tooth discoloration reported in the clinical application of bismuth oxide-containing hydraulic cements. The present study examined the effects of this experimental cement (Quick-Set2) on the viability and proliferation of human dental pulp stem cells (hDPSCs) by comparing the cellular responses with commercially available calcium silicate cement (white mineral trioxide aggregate; WMTA) after different aging periods. Cell viability and proliferation were examined using assays that examined plasma membrane integrity, leakage of cytosolic enzyme, caspase-3 activity for early apoptosis, oxidative stress, mitochondrial metabolic activity and intracellular DNA content. Results of the six assays indicated that both Quick-Set2 and WMTA were initially cytotoxic to hDPSCs after setting for 24 h, with Quick-Set2 being comparatively less cytotoxic than WMTA at this stage. After two aging cycles, the cytotoxicity profiles of the two hydraulic cements were not significantly different and were much less cytotoxic than the positive control (zinc oxide–eugenol cement). Based on these results, it is envisaged that any potential beneficial effect of the discoloration-resistant calcium aluminosilicate cement on osteogenesis by differentiated hDPSCs is more likely to be revealed after outward diffusion and removal of its cytotoxic components.

Calcium silicate cements have been widely used for treatment of non-surgical and surgical endodontic conditions, including vital pulp therapy^1^. Similar to the ordinary Portland cements used in the building industry, these cements contain lime and silica as the principal oxides, in the form of tricalcium and dicalcium silicate. Minor amounts of tricalcium aluminate, calcium sulfate and calcium aluminoferrite (dark phase) are also present^2^. On reaction with water, these hydraulic cements produce amorphous calcium silicate hydrate and crystalline calcium hydroxide as the principal hydration phases that bind the unreacted mineral particles together to form a conglomerated structure^3^. Calcium silicate cements designed for biomedical uses are biocompatible, bioactive, and possess clinically acceptable sealing properties and the ability to induce reparative hard tissue formation^2^. Their limitations include suboptimal handling characteristics, long setting times, washout during setting, minimal adhesion to canal wall dentin and relatively high solubility in moist environment^2^. Although some of those undesirable attributes have been addressed in more recent formulations^4^, none of the currently available cements addresses all of the aforementioned challenges. The major shortcoming of calcium silicate cements is that they do not set optimally in acidic environments^5^. These cements are also vulnerable to attack by acids and calcium-chelating irrigants because the calcium hydroxide phase is rapidly dissolved by those agents, thereby increasing the porosity of the set cements^6^.

Calcium aluminate cements were developed in the late 19^th^ century as an alternative to calcium silicate-base cements^6^. These cements emerged from the motivation to develop cements which are resistant to acid attack, and biogenic corrosion by acids produced by acidogenic bacteria^7^. Although calcium aluminate cements are also designated as hydraulic cements, they differ from calcium silicate cements in the nature of the active phase that leads to setting and hardening. Calcium aluminate cements contain lime and alumina as the principal oxides, with little or no silica^8^. The oxides combine to give monocalcium aluminate as the principal active phase, which reacts with water to release calcium and hydroxyl ions. This is followed by precipitation of alumina hydrate and various forms of temperature-dependent calcium aluminate hydrates. Calcium aluminate cements are less basic (pH ~10) than tricalcium silicate cements after setting^8^. They are more acid-resistant because alumina hydrate is stable down to pH ~3–4. Dissolution of calcium aluminate hydrate also leads to the formation of additional alumina hydrate. The latter fills in pores and protects the set cement from further acid attack^6^,^9^.

Because of their potential resistance to dissolution by acidogenic bacteria derived from oral plaque biofilms and their potential bioactivity, calcium aluminate cements have been used as restorative materials (Doxadent; Doxa Dental AB, Uppsala, Sweden)^10^, and for crown and bridge cementation when combined with glass ionomers (Ceramir C&B, Dosa Dental AB)^11^. Although the clinical performance of the luting cement was satisfactory^11^, filling materials prepared from calcium aluminate cements exhibited unacceptable failure rates when used for stress-bearing restorations^12^. Since calcium aluminate cements release calcium and hydroxyl ions necessary for precipitation of carbonated apatite and stimulation of hard tissue regeneration, they have also been advocated for use in endodontics, with the same indications as calcium silicate cements.

Incorporation of calcium aluminate in root canal cement was first reported in 1991^13^. A set of endodontic cements was subsequently developed based on the formulation patented by Pandolfelli *et al.*^14^. This formulation has the composition (in weight %): Al_2_O_3_ (≥68.0), CaO (≤31.0), SiO_2_ (0.3–0.8), MgO (0.4–0.5) and Fe_2_O_3_ (<0.3), and incorporates additional rheology modifiers and radiopaque additives. The cement contains calcium monoaluminate and calcium dialuminate as the active mineral phases responsible for the hydraulic setting reaction. Impurities such as Fe_2_O_3_ and MgO were reduced to minimize the potential for tooth darkening and undesirable water-induced expansion. Marketed as EndoBinder (Binerware, São Carlos, SP, Brazil)^15^, the calcium aluminate cement has been advocated as an alternative to calcium silicate endodontic cements based on its biocompatibility^15^, minimal stimulation of inflammatory reactions in animal studies^16^, as well as its ability to promote repair of mineralized tissues in cell culture studies^17^.

Hybrid aluminosilicate cements have been developed based on the premise that silicate phases are desirable in dentinogenesis and osteogenesis for healing of pulpal and periapical tissues^18^. Two formulae of calcium aluminosilicate cements have been tested for their potential use in endodontics, which were designated as Capasio^19^ and Quick-Set (Primus Consulting, Bradenton, FL, USA)^20^,^21^. Apart from the inclusion of silicate in the cement formulations, these materials differ from Endobinder in that the water-based liquid components do not contain salts for acceleration of setting reactions, but proprietary water-soluble polymers and other suspending agents^22^. The setting time of a commercial version of calcium silicate cement (white ProRoot MTA, Dentsply Tulsa Dental Specialties, Tulsa, OK, USA; commericalized calcium silicate cement) is 150 minutes, while that of Capasio (experimental aluminosilicate cement) is no more than 15 minutes^21^.

A prerequisite of hydraulic cements designed for dental applications is the incorporation of radiopaque additives. This enables the cements to be identified by radiography. Bismuth oxide is the most common radiopaque filler employed in calcium silicate, calcium aluminate and calcium aluminosilicate cements because of its high atomic number and consequently high opacity to X-rays. However, tooth discoloration has been documented in clinical studies when endodontic procedures were performed with calcium silicate cements which do not contain calcium aluminoferrite (i.e. white mineral trioxide aggregate)^23^,^24^. Discoloration of calcium aluminoferrite-free calcium silicate cements containing bismuth oxide as radiopaque fillers occurs from white to gray, dark brown or black, after the set cements were exposed to sodium hypochlorite^25^,^26^, chlorhexidine gluconate^26^, and after contact with tooth structure^27^, blood^28^ or formaldehyde^29^. Although bismuth oxide-induced discoloration does not affect the stability or radiopacity of the set cements, the color change is disconcerting when these cements are used for coronal restorations within the esthetic zone. Alternative oxides with variable degrees of radiopacity (e.g. zirconium oxide) have been incorporated in experimental tricalcium silicate^30^ and calcium aluminate cements^31^. To circumvent the problem of tooth discoloration in calcium aluminosilicate cements, an experimental discoloration-resistant calcium aluminosilicate cement has been formulated (Quick-Set2; Primus Consulting, Bradenton, FL, USA) by replacing the bismuth oxide radiopacifier with tantalum oxide. In addition, free alumina has been eliminated to increase the hydraulic phase percentage.

Hydraulic cements placed in closed proximity with pulpal and periradicular tissues have to be biocompatible to expedite reparative dentinogenesis by pulpal stem cells during vital pulp therapy. Thus, the objective of the present study was to examine the effects of the experimental discoloration-resistant calcium aluminosilicate cement on the viability and proliferation of human dental pulp stem cells (hDPSCs) prior to their differentiation. Although hDPSCs are multipotent and have the capacity to differentiate into chondrogenic, adipogenic and osteogenic cells, the well-being of the original stem cells is a prerequisite for these events to occur^32^,^33^. The null hypothesis tested was that there is no difference in the various facets of cytotoxicity induced by the experimental calcium aluminosilicate cement and a calcium silicate endodontic cement when these set cements are placed in close proximity with undifferentiated hDPSCs.

## Materials and Methods

### Specimen preparation

Two hydraulic cements were tested: Quick-Set2 and white ProRoot MTA (WMTA), the latter being a bismuth oxide-containing calcium silicate cement. For each cement, the powder was mixed with the proprietary hydrogel or deionized water, according to the instructions of the respective manufacturer, using a liquid/powder ratio of 0.3. The mixed materials were placed in pre-sterilized Teflon molds (5-mm diameter and 3-mm thick), covered with pre-sterilized Mylar sheets, and allowed to set in a 100% humidity chamber for 24 h. Disks of similar dimensions were prepared from Intermediate Restorative Material (IRM; Dentsply Caulk, Milford, DE, USA), a zinc oxide–eugenol cement, which were assigned as the positive control. For the negative control, the hDPSCs (described below) were not exposed to any material. All set materials were sterilized with ultraviolet light for 4 h prior to testing.

### Resistance to discoloration

Two disks prepared from each hydraulic cement were used to examine the potential of the set materials to resist discoloration. Each disk was incubated in 5 mL of one of the following solutions at 37 °C for 7 days: deionized water, 2% chlorhexidine gluconate (Clorox Healthcare^TM^, Oakland, CA, USA), 8.25% sodium hypochlorite (Clorox^®^ Germicidal Bleach, Clorox Healthcare^TM^) and 10% neutral buffered formaldehyde solution (Sigma-Aldrich, St. Louis, MO, USA). The materials and solutions were kept in the dark during incubation. After 7 days, the material disks were retrieved, rinsed with deionized water, air-dried and photographed.

### Cell culture

Human dental pulp stem cells were used in the present cell culture study. Pulpal tissues were obtained from non-carious third molars extracted from young healthy patients (18–25 years old) according to a protocol approved by the Ethics Committee of the Fourth Military Medical University. The informed consent was obtained from all subjects. The dental pulps were minced and digested in a solution containing 3 mg/mL type I collagenase and 4 mg/mL dispase (Gibco BRL, Gaithersburg, MD, USA) at 37 °C for 2 h. Single-cell suspensions were obtained by passing the cells through a 70-mm strainer (BD Falcon, Franklin Lakes, NJ, USA) and cultured in growth medium (α-modified Eagle medium (Gibco) supplemented with 10% fetal bovine serum (Gibco), 100 units/mL penicillin and 100 mg/mL streptomycin) in 5% CO_2_ at 37 °C. To identify hDPSCs, the cultured cells were incubated with fluorescent dye-conjugated monoclonal antibodies for different Cluster of Differentiation (CD) cell-surface molecular markers, including anti-CD29, anti-CD34, anti-CD44, anti-CD45, anti-CD90 and anti-CD105 (EMD Millipore Corp., Billerica, MA, USA), and sorted using a flow cytometer (Elite ESP, Beckman Coulter, Fullerton, CA, USA)^32^,^33^. To confirm the specificity of primary antibody binding nonspecific mouse IgM isotype control (lambda monoclonal MOPC-104E, Abcam, Cambridge, MA, USA) which matches the primary antibody’s host species was substituted for the primary antibody. The CD90^+^/CD105^+^/CD45^−^/CD34^−^ hDPSCs were sorted, collected and expanded. Sorted hDPSCs from the third to sixth passages were used for subsequent experiments.

The multipotency of the hDPSCs was tested using chondrogenic, adipogenic and osteogenic culturing conditions^34^. For chondrogenic differentiation, 1.0 × 10^6^ hDPSCs were cultured until they achieved 60–70% confluency. The cells were centrifuged at 800 rpm for 6 min to form a pellet. After incubation in complete growth medium for 24 h, the pellet was incubated in chondro-inductive medium, consisting of high-glucose Dulbecco’s Modified Eagle Medium (Gibco) containing 0.1 mM dexamethasone, 50 mg/mL L-ascorbic acid-2-phosphate (Sigma-Aldrich, St. Louis, MO, USA), 40 mg/mL of L-proline (Sigma-Aldrich), 1% insulin-transferring selenium (ITS + premix; 100X; Sigma-Aldrich), 15% fetal bovine serum, 10 ng/mL transforming growth factor-β1 (R&D Systems, Minneapolis, MN, USA) and 2% Antibiotic-Antimycotic (Life Technologies, Thermo Scientific, Carlsbad, CA, USA). After 4 weeks of chondrogenic induction, the specimens were fixed with 4% paraformaldehyde, stained with Alcian blue (Lifeline Cell Technology, Frederick, MD, USA) and examined by light microscopy (Leica DM 2500,Wetzlar, Germany). For adipogenic differentiation, 3 × 10^5^ hDPSCs were cultured in adipo-inductive medium supplemented with 0.5 mM methylisobutylxanthine, 0.5 mM hydrocortisone and 60 μM indomethacin (all from Sigma-Aldrich) and 2% Antibiotic-Antimycotic for four weeks. The cultured cells were fixed with 4% polyoxymethylene, stained with 0.3% Oil Red O solution (Sigma-Aldrich) and examined by light microscopy. For osteogenic differentiation, 3 × 10^5^ hDPSCs were cultured in osteo-inductive medium supplemented with 100 nM dexamethasone, 0.2 mM ascorbic acid-2-phosphate and 10 mM β-glycerophosphate (all from Sigma-Aldrich) and 2% Antibiotic-Antimycotic. After culturing in osteogenic medium for four weeks, the cells were fixed with 4% paraformaldehyde and stained with 1% Alizarin red S for light microscopy examination of mineralized nodules.

### Cyclic aging protocol

Because cytotoxic components present in hydraulic cements can diffuse out of the material, the effects of materials on the viability and proliferation of the stem cells were evaluated using a cyclic aging protocol^35^. A weekly cycle consisted of evaluating the toxicity of the materials after they were placed in the vicinity of the stem cells for 3 days. Freshly mixed hydraulic cement disks that had been set for 24 h were used for the 1^st^ cycle. After the first testing cycle, the cement disks were retrieved and re-immersed in sterilized deionized water for 4 days to enable potentially toxic components to diffuse out of the disks. The same disks were used for testing during the next weekly cycle. Three cycles were used for the evaluations described in the subsequent sections.

### Cell viability assays

#### Membrane integrity

Flow cytometry was employed with a differential staining technique for sorting and counting of individual cells within a cohort of hDPSCs that expressed changes in plasma membrane permeability induced by the toxicity of the materials. The hDPSCs were plated in 6-well plates at a density of 10^5^ cells/cm^2^ and incubated at 37 °C in a humidified 5% CO_2_ atmosphere for 24 h. Materials were tested in three cycles as previously described. For each cycle, the cement and positive control disks were placed individually in Transwell inserts with a 3-μm pore size (BD Falcon, Franklin Lakes, NJ, USA) to prevent direct contact of the cells by the specimen. After the insert was placed over the plated cells, an additional 2 mL of complete growth medium was added to each well to ensure that the level of the culture medium was above the sides of the Transwell insert. The disks were exposed to the plated cells for 3 days without further change in culture medium. The same procedures were adopted for the negative control, with the exception that no material was placed inside the Transwell insert.

After exposure to the materials, the stem cells were detached from the culture wells with trypsin, and re-suspended at 2 × 10^6^ cells/mL in the binding buffer included in the Apoptosis and Necrosis Quantification Kit (Biotium Inc, Hayward, CA, USA). The cells were stained with FITC-Annexin V (AnV; λ_abs_/λ_em_ = 492/514 nm) and ethidium homodimer III (Etd-III; λ_abs_/λ_em_ = 528/617 nm) as the fluorescence stains for cytoplasmic membrane phospholipids and nucleic acids, respectively. During apoptosis, phosphatidylserine is translocated from the inner to the outer surface of the cell for phagocytic cell recognition^36^. Human anti-coagulant Annexin V is a 35 kDa, Ca^2+^-dependent phospholipid-binding protein with a high affinity for phosphatidylserine. Annexin V labeled with fluorescein (FITC) can identify apoptotic cells by binding to phosphatidylserine exposed on the outer leaflet of the cytoplasmic membrane, which results in the expression of green fluorescence within the cytoplasm. Necrosis is usually caused by a severe cellular insult, resulting in the loss of nuclear membrane integrity. Ethidium homodimer III is a highly positive-charged nucleic acid probe, which is impermeable to live cells or apoptotic cells, but stains necrotic cells with red fluorescence. The stained hDPSCs were sorted with a FACSCalibur flow cytometer (BD Biosciences, San Jose, CA, USA) to determine the percent distribution of healthy (AnV/Etd-III negative), early apoptotic (AnV positive, Etd-III negative), late apoptotic (AnV/Etd-III positive) and necrotic cells (AnV negative, Etd-III positive). The experiment was performed in sextuplicates.

Fluorescent microscopy was used to document fluorescence associated with alterations in membrane permeability of the hDPSCs. The cells were plated on glass cover slips at a density of 400 cells/cm^2^. Cell-plated cover slips were placed inside 6-well plates for culture and the stem cells were allowed to establish for 24 h. The materials were tested 24 h after mixing (1^st^ cycle) and after aging for 2 weeks (3^rd^ cycle). After exposure to the materials for 3 days, the hDPSCs were triple-stained with AnV (green-fluorescence), Etd-III (red-fluorescence) and Hoechst 33342, a cell-permeable blue-fluorescent bis-benzimide nucleic acid counterstain that emits blue fluorescence (λ_abs_/λ_em_ = 350/461 nm) when bound to double-stranded DNA in both viable and non-vital cells. Stained hDPSCs were examined with a fluorescent microscope (Axioplan 2, Carl Zeiss, Oberkochen, Germany).

#### Leakage of cytosolic enzyme

Cells that have compromised plasma membrane integrity release lactate dehydrogenase (LDH) into the surrounding medium. This cytosolic enzyme catalyzes the conversion of lactate to pyruvate via reduction of NAD^+^ (oxidized form of nicotinamide adenine dinucleotide) to NADH. The loss of intracellular LDH and its release into the culture medium is a biomarker for irreversible cell membrane damage induced by the toxicity of materials^37^. Quantitation of LDH activity was performed with the Pierce^TM^ LDH Cytotoxicity Assay Kit (Thermo Scientific). Briefly, hDPSCs were plated in 24-well plates at a density of 10^4^ cells/cm^2^ and exposed to cement disks derived from the three aging cycles. For each cycle, the disks were exposed to the plated cells for 3 days without further change in culture medium. At the designated time, the LDH released into the culture medium was transferred to a new plate and mixed with Reaction Mixture. After incubation for 30 min, the reaction was stopped by adding Stop Solution. Absorbances at 490 nm and 680 nm were measured using a microplate reader (Synergy HT, BioTek Instruments, Winooski, VT, USA). The LDH activity was determined by subtracting the 680 nm background absorbance from the 490 nm absorbance. For controls, additional hDPSCs were used for testing the spontaneous LDH activity in sterile ultrapure water, and for testing the maximum LDH activity of the cells by exposing them to 10X Lysis Buffer. The experiment was performed in sextuplicates. Percent cytotoxicity was calculated using the formula: [(Material-mediated LDH activity–Spontaneous LDH activity)/(Maximum LDH activity–Spontaneous LDH activity)] ×100.

#### Caspase-3 activity

Caspase-3, a member of the cysteine-requiring aspartate protease family, plays a crucial role in mediating intracellular events associated with apoptosis, including chromatin condensation, DNA fragmentation and cell blebbing^38^. The caspase-3 activity of hDPSCs exposed to different cements was determined with a Caspase-3 Colorimetric Assay Kit (Sigma-Aldrich). Briefly, after hDPSCs (10^5^ cells/cm^2^) were exposed to materials from the three aging cycles for 3 days each, they were lysed with Lysis Buffer for 15 min. The cell lysates were allowed to react with a p-nitroanilide (pNA) conjugated peptide substrate for 90 min. Hydrolysis of the peptide substrate by the caspase-3 present in the cell lysate led to the release of p-NA, the absorbance of which was recorded at 405 nm. The concentration of the released p-NA was determined from a calibration curve prepared with different concentrations of a p-NA standard. The experiment was performed in sextuplicates.

#### Oxidative stress

Redox homeostasis is dynamically regulated within cells because there is a narrow concentration range that governs whether reactive oxygen species (ROS) induce toxicity or act as second messengers for redox signaling in cell proliferation, differentiation, apoptosis or autophagy^39^. Oxidative stress reflects an imbalance between ROS production and the cell’s ability to detoxify the reactive intermediates and repair damage. Excessive ROS production causes toxic effects through the production of peroxides and free radicals that damage cellular components such as proteins, lipids and DNA. Hence, evaluation of intracellular ROS formation provides another perspective for evaluating the cellular responses to the hydraulic cements. Detection of intracellular ROS in hDPSCs was performed using the CellROX^®^ Orange Oxidative Stress Reagent (Life Technologies, Thermo Fisher). After the cells (10^5^ cells/cm^2^) were exposed to test materials from the three aging periods for 3 days each, they were detached, centrifuged and re-suspended in 1% phosphate-buffered saline. CellROX^®^ Orange (a fluorescent redox cytoplasmic stain; λ_abs_/λ_em_ = 545/565 nm) was added to the cells at a final concentration of 5 μM and incubated at 37 °C for 30 min. The FACSCalibur flow cytometer was used to detect the percentage of ROS-positive cells for each material/aging period. The experiment was performed in sextuplicates. Additional cells were plated on coverslips, double-stained with CellROX^®^ Orange and Hoechst 33342, and examined with a fluorescent microscope (Axioplan 2) for qualitative evaluation of intracellular ROS distribution.

### Cell Proliferation Assays

#### Metabolic activity

The mitochondrial activity of hDPSCs after their exposure to the materials from the three aging cycles was evaluated using 3-(4,5-dimethylthiazol-2-yl)-2,5-diphenyltetrazolium bromide (MTT) assay. This assay measured cell metabolic activity based on the rationale that dead cells are incapable of metabolizing tetrazolium salts via mitochondrial dehydrogenases involved in the citric acid cycle and the electron transport chain^40^. The hDPSCs were plated in 24-well plate at a density of 10^4^ cells/cm^2^ and incubated for 24 h. The assay was performed by incubating the hDPSCs with MTT-succinate solution for 60 min and then fixing the cells with Tris-formalin. The purple MTT formazan produced in the cells was dissolved *in-situ* using dimethyl sulfoxide-NaOH. The optical density of the formazan was measured at 562 nm. The optical density of blank dimethyl sulfoxide-NaOH was subtracted from all wells. The formazan content of each well was computed as a percentage of the mean of the unexposed hDPSC negative control.

#### Cellular DNA content

The effect of test materials on the proliferation of hDPSCs was examined using the CyQUANT Cell Proliferation Assay Kit (Life Technologies, Thermo Fisher). The assay utilizes fluorometry for indicating cell numbers, based on the fluorescence exhibited by the binding of CyQUANT GR cyanine dye to cellular nucleic acids^41^. Briefly, the hDPSCs were plated in 24-well plates (10^4^ cells/cm^2^), cultured for 24 h and exposed to the materials derived from the three aging cycles for 3 days each. After removal of the materials, the cells were exposed to the CyQUANT GR dye/cell lysis buffer for 5 min. The absorbance of the cell lysate was determined at λ_abs_/λ_em_ = 480/530 nm using a fluorescence microplate reader (FL600, BioTek Instruments, Winooski, VT, USA). The concentration of DNA (in ng/μL) was calculated using a pre-established standard curve that correlates fluorescence intensity with known DNA concentrations.

### Statistical analyses

Data obtained from each assay were analyzed separately to examine the effects of “material” and “aging cycle”, and the interaction of these two factors on the parameter investigated, using two-factor repeated measures analysis of variance (ANOVA). Because the IRM positive control group was included for identifying the discriminatory potential of each assay, data from this group were excluded to increase the robustness of the statistical analyses. Post-hoc comparisons were performed using Holm-Sidak procedures. Each data set was first evaluated for its normality (Shapiro-Wilk test) and equal variance (modified Levene test) assumptions. When those assumptions were violated, the respective data set was non-linearly transformed to satisfy those assumptions prior to the use of parametric statistical procedures. Statistical significance for all tests was set at α = 0.05.

## Results and Discussion

**Figure 1:**
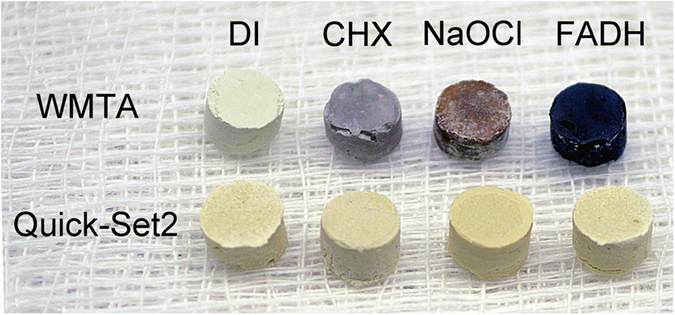
Resistance to discoloration. Color changes in white ProRoot MTA (WMTA) and Quick-Set2 after the set materials were incubated in deionized water (DI), 2% chlorhexidine (CHX), 8.25% sodium hypochlorite (NaOCl) and 10% formaldehyde (FADH) for 7 days.

Color changes after the set hydraulic cements were incubated in different solutions (Fig. 1) is an indirect evidence for the possibility of tooth discoloration caused by the material. The color of white ProRoot MTA (WMTA) remained stable after immersion in deionized water. Although no spectrophotometric methods were employed, color changes were intense enough to be visibly discerned after WMTA were incubated in the other solutions; specimens turned gray after immersion in 2% chlorhexidine, dark brown after immersion in 8.25% sodium hypochlorite and black after immersion in 10% formaldehyde. These color changes are similar to what had been reported in the literature for calcium silicate cements that utilized bismuth oxide as radiopacifier^25^,^26^,^29^. Sodium hypochlorite and chlorhexidine are commonly used irrigants in root canal treatment. Although there is no indication for formaldehyde in clinical practice, WMTA root end fillings performed in animal studies turned black after fixation with formaldehyde during preparation of histological sections (Primus, unpublished results). In comparison, Quick-Set2 was resistant to discoloration after the specimens were incubated in the different solutions. The results suggest that tantalum oxide may be a better alternative than bismuth oxide as a color-stable radiopacifier for hydraulic cements that are designed for clinical procedures involving the coronal aspect of a tooth.

The use of animal surrogates for toxicology testing in the European cosmetics industry has been banned since March 2013^42^. Prior to this implementation, the U.S. National Academy of Sciences has published a consensus report in 2007, entitled “Toxicity Testing in the 21st Century: A Vision and a Strategy”^43^. The report envisioned a paradigm shift in future toxicity testing by transitioning from current expensive and lengthy *in vivo* animal testing with qualitative endpoints, to *in vitro* toxicity assays on human cells or cell lines using a suite of toxicity pathway assays with quantitative parameters^44^. This report supported the use of human stem cells, cultured *in vitro*, as critical target cells in future toxicology testing^45^,^46^. Dental pulp stem cell is one of the five dental-tissue derived stem/progenitor cells that possess mesenchymal stem cell-like properties, including the capacity to regenerate dental hard tissues^47^. Undifferentiated hDPSCs were used in the present work because these cells are likely to be involved when hydraulic cements are placed over exposed human dental pulps in direct pulp capping or pulpotomy procedures. Although non-human cell lines had been used almost exclusively in previous cytotoxic studies of hydraulic cements, human cells can predict the response of the human body to chemicals more accurately than animal cells. Due to species differences, humans and animals respond differently to chemical exposures, with concordance rates of approximately 63% for using nonrodent animal models, and only 43% for using rodent models for prediction of human toxicity^48^. These relatively low concordance rates point to the need for employing cells of human origin for toxicology testing; another advantage of using human cells is their potential to reveal human susceptibility factors for toxicity^46^.

**Figure 2:**
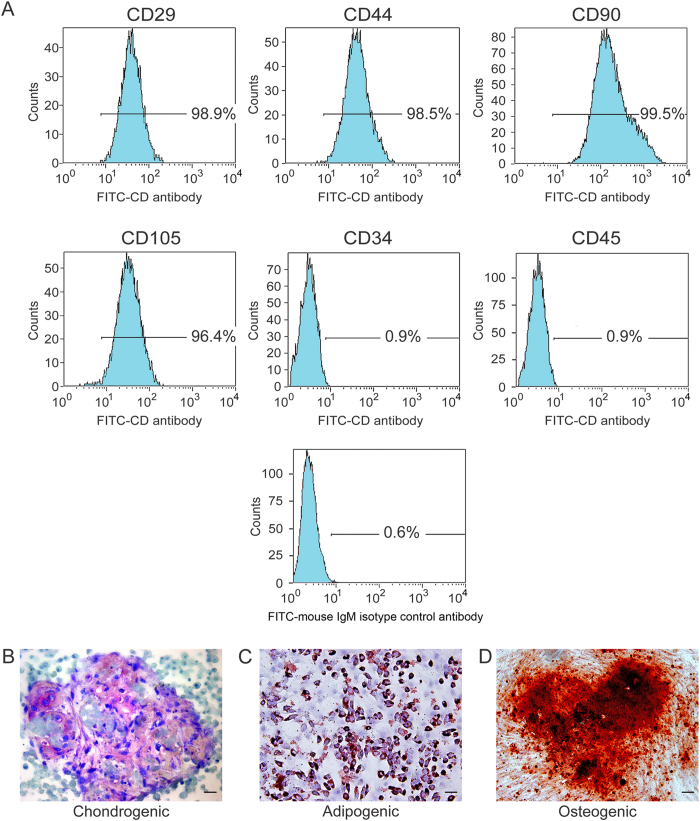
Immunophenotypic and multipotency characteristics of hDPSCs. (**A**) Immunophenotyping of hDPSCs using FITC dye-conjugated antibodies to identify different Cluster of Differentiation (CD) cell-surface molecules. FITC dye-conjugated mouse IgM antibody was used as isotype control. The weak staining of the isotype IgM control antibody is indicative of the specificity of binding of the CD antibodies. (**B**) Cartilage extracellular matrix formed by the differentiated cells after incubation in chondrogenic medium (stained with Alcian blue). (**C**) Intracellular lipid vacuoles that were found in differentiated cells after incubation in adipogenic medium (stained with Oil Red O). (**D**) Mineralized nodules formed by differentiated cells after incubation in osteogenic medium (stained with Alizarin red S). For (**B**–**D**)bar = 50 μm.

Consistent with other mesenchymal stem cell populations, the majority of hDPSCs exhibited intense expressions of mesenchymal surface molecular markers (CD29–98.9%, CD44–98.5%, CD90–99.5% and CD105–96.4%). The hDPSCs also exhibited weak expressions of surface markers for hematopoietic system-derived cells (CD34–0.9% and CD45–0.9%) (Fig. 2A). Weak staining of the mouse IgM isotype control antibody confirmed the specificity of primary antibody binding (Fig. 2A). The multipotent nature of the hDPSCs was confirmed by the observation that these cells have the potential to develop into chondrocytes after chondrogenic induction, with the formation of a cartilaginous-like extracellular matrix (Fig. 2B). Following adipogenic induction, intracellular lipid vacuoles could be identified within the differentiated adipocytes (Fig. 2C). Mineralized nodules that were stained with Alizarin red S appeared in the extracellular milieu after the hDPSCs were cultured in osteogenic differentiation medium (Fig. 2D).

**Figure 3:**
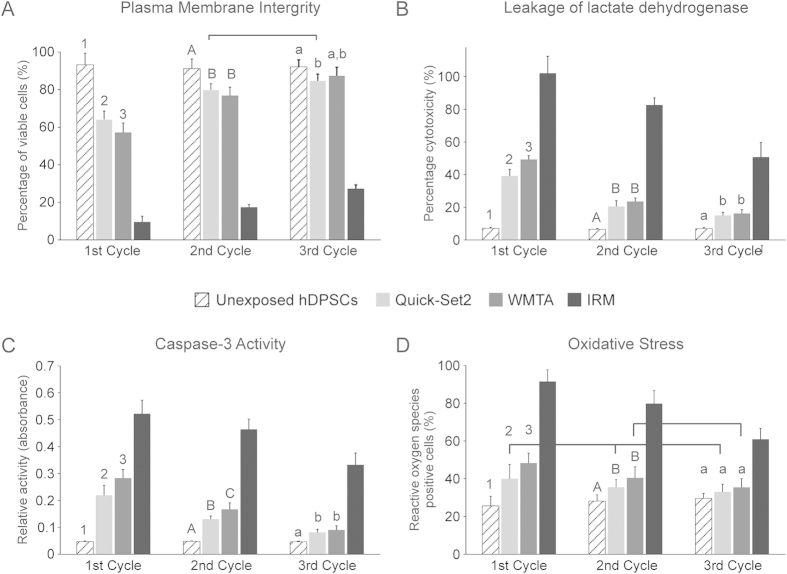
Results of cell viability assays after the hDPSCs were exposed to materials derived from the 3 aging cycles. (**A**) Membrane integrity of hDPSCs after the cells were stained with FITC-Annexin V and ethidium homodimer III. Chart represents the percentages of healthy hDPSCs that were not stained by Annexin V and ethidium homodimer III. (**B**) Leakage of lactate dehydrogenase from hDPSCs that had compromised plasma membrane permeability. (**C**) Caspase-3 activity of hDPSCs as an indicator of cell apoptosis. (**D**) Expression of reactive oxygen species from hDPSCs as an indicator of intracellular oxidative stress. Statistical analyses were only conducted for hDPSCs exposed to the two hydraulic cements and unexposed hDPSCs (negative control). For the factor “material” in each chart, groups labeled with the same designators (numerals for 1^st^ cycle, upper case letters for 2^nd^ cycle and lower case letters for 3^rd^ cycle) are not significantly different (P > 0.05). For the factor “aging cycle” in each chart, cycles from the same hydraulic cement that are connected with a horizontal bar are not significantly different (P > 0.05). For unexposed hDPSCs, there are no differences in activities among the 3 cycles (P > 0.05; horizontal bar not shown).

Cells exposed to toxic materials may result in a variety of cell fates. Depending on the toxicity level, the cells may undergo necrosis, in which they lose membrane integrity and die from cell lysis. Alternatively, the cells may activate a genetic program of controlled cell death (apoptosis). They can also stop active growth and cell division (decrease in cell viability and proliferation). In addition, cytotoxic materials may reduce intracellular production of antioxidants or enhance mitochondrial production of ROS, which, in turn, augment the level of intracellular oxidative stress that adversely affect cell proliferation. Results of the four cell viability assays are shown in Fig. 3. Figure 3A represents the percentage of healthy cells with intact plasma membranes that were present within a consortium of hDPSCs after their exposure to different materials that had been aged for 3 cycles. Two-factor repeated measures ANOVA comparing hDPSCs exposed to the two hydraulic cements with unexposed hDPSCs indicated that the type of material (P < 0.001), aging cycle (P < 0.001) and the interaction of these two factors (P < 0.001) all had significant influences on the percentage of healthy hDPSCs that were non-permeable to the two fluorescence stains for cytoplasmic membrane phospholipids and nucleic acids. Post-hoc pairwise comparisons (only comparisons with significant differences are described) showed that for the factor “aging cycle” within materials, the numbers of healthy cells in the 1^st^ cycle of Quick-Set2 was lower than those in the subsequent two cycles. For WMTA, the number of healthy hDPSCs in each cycle was higher than the subsequent cycle, in the order: 1^st^ cycle < 2^nd^ cycle < 3^rd^ cycle. For the factor “material” within the 1^st^ cycle, the number of healthy unexposed hDPSCs was higher than those exposed to Quick-Set2 or WMTA, while the number of healthy cells in Quick-Set2 was higher than WMTA. For the factor “material” within the 2^nd^ cycle, the number of healthy unexposed hDPSCs was higher than Quick-Set2 or WMTA. For the factor “material” within the 3^rd^ cycle, significant difference in the number of healthy cells was only observed between unexposed hDPSCs and hDPSCs exposed to Quick-Set2.

**Figure 4:**
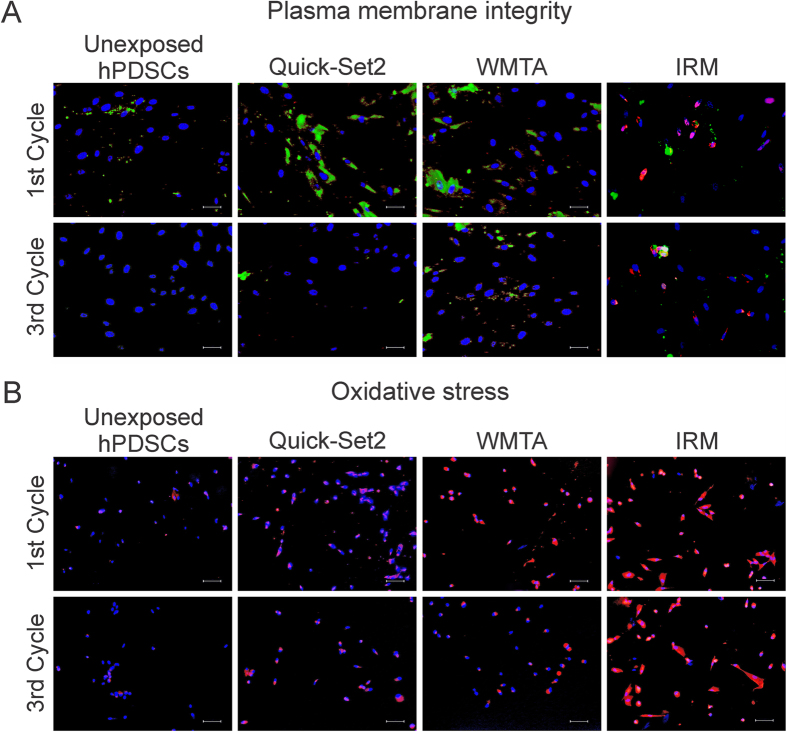
Fluorescent microscopic evaluation of the plasma membrane integrity and oxidative stress after the hDPSCs were exposed to materials derived from the 1^st^ and 3^rd^ aging cycle. (**A**) Fluorescent microscopy images of hDPSCs that were triple-stained with Hoechst 33342 (blue fluorescent nuclear counterstain), ethidium homodimer III (red fluorescent non-vital DNA dye) and FITC-Annexin V (green fluorescent phosphatidylserine-binding cytoplasmic dye). Healthy cell nuclei were stained blue. Apoptotic cells showed green cytoplasm and blue nuclei. Necrotic cells showed red or pink nuclei. Dead cells progressing from the apoptotic cell population were stained green, red and blue. Bars = 25 μm. (**B**) Fluorescent microscopy images of hDPSCs double-stained with CellROX^®^ Orange and Hoechst 33342. Cytoplasms of cells exhibiting oxidative stress were stained orange. Bars = 50 μm.

Images acquired by fluorescent microscopy were complementary of the flow cytometry results (Fig. 4a). Unexposed hDPSCs from the 1^st^ or 3^rd^ cycle were predominantly healthy and exhibited blue-fluorescent nuclei with minimal signs of apoptosis or necrosis. Cells exposed to the IRM positive control were mostly apoptotic or necrotic, with prevalence of green fluorescent cytoplasm that could be attributed to apoptosis. Occasionally, the cells exhibited partially red-fluorescent cytoplasm (caused by leaching of nucleic acid components) or pink nuclei (merging of blue and red fluorescence) that are characteristic of necrosis or cell death progressing from apoptosis. Stem cells that were exposed to Quick-Set2 or WMTA in the 1^st^ cycle were predominantly healthy; nevertheless, cells with green-fluorescent cytoplasm could be observed. The number of apoptotic cells that exhibited green-fluorescent cytoplasm was substantially reduced after hDPSCs were exposed to Quick-Set2 or WMTA from the 3^rd^ cycle.

Unlike complex living systems, cultured eukaryotic cells undergoing apoptosis eventually die by secondary necrosis^49^. Because features of apoptosis and necrosis overlap in cell cultures, two or more assays are necessary to confirm that cell death occurs via apoptosis. This prompted the examination of LDH leakage and caspase-3 activity of the hDPSCs in the present study. For LDH leakage (Fig. 3B), the type of material (P < 0.001), aging cycle (P < 0.001) and the interaction of these two factors (P < 0.001) all had significant influences on leakage of the cytosolic enzyme from hDPSCs. For pairwise comparisons of the factor “aging cycle” within materials (only comparisons with significant differences are described), LDH leakage from hDPSCs continued to decrease after each aging cycle for both hydraulic cements. For the factor “material” within the 1^st^ aging cycle, LDH leakage was lower for the unexposed hDPSCs compared with those exposed to the two hydraulic cements; LDH leakage in Quick-Set2 was also lower than WMTA. For the factor “material” within the 2^nd^ and 3^rd^ cycles, LDH leakage was still lower for the unexposed hDPSCs when compared with the two hydraulic cements.

For caspase-3 activity (Fig. 3C), the type of material (P < 0.001), aging cycle (P < .001) and the interaction of these two factors (P < 0.001) all had significant influences on the production of this enzyme during the early stage of apoptosis by hDPSCs. For pairwise comparisons of the factor “aging cycle” within materials (only comparisons with significant differences are described), caspase-3 activity by hDPSCs continued to decrease after each aging cycle for both hydraulic cements. For the factor “material” within the 1^st^ and 2^nd^ cycles, caspase-3 activity for the unexposed hDPSCs was lower than the two hydraulic cements; enzyme activity in cells exposed to Quick-Set was also lower than WMTA. For the factor “material” in the 3^rd^ cycle, caspase-3 activity for the unexposed hDPSCs was still significantly lower than hDPSCs exposed to Quick-Set2 or WMTA.

Oxidative stress is created when a cell’s metabolic prooxidant production exceeds its antioxidant capacity^50^. Approximately 2% of the oxygen consumption by cells is utilized by the mitochondria for ROS production^51^. When ROS production is low, damage of critical intracellular biomolecules can be handled by antioxidant enzymes produced by the cells. Excessive ROS in the form of hydrogen peroxide, superoxide or hydroxyl radical can react with cellular DNA, proteins and lipids to produce other radicals or cytotoxic products that lead to cell damage. Figure 4B shows images of hDPSCs stained by CellROX^®^ Orange and the Hoechst 33342 nuclear counterstain. Cells with increased levels of oxidative stress exhibited orange fluorescence in their cytoplasm, which was most notable in the IRM positive control groups. The chart in Fig. 3D represents the percentage of ROS-positive hDPSCs after exposure to the materials. The type of material (P < 0.001), aging cycle (P = 0.022) and the interaction of these two factors (P = 0.013) were found to significantly affect the levels of oxidative stress in unexposed hDPSCs or hDPSCs exposed to the two hydraulic cements from the three cycles. For the factor “aging cycle” within materials (only pairwise comparisons with significant differences are described), the level of oxidative stress in hDPSCs exposed to WMTA from the 1^st^ cycle was higher than the 2^nd^ and 3^rd^ cycles. For the factor “material” within the 1^st^ cycle, there were less ROS-positive cells in unexposed hDPSCs, compared to hDPSCs that had been exposed to the two hydraulic cements, and less ROS-positive cells in Quick-Set-2 compared to WMTA. For the factor “material” within the 2^nd^ cycle, the percentage of ROS-positive cells in unexposed hDPSCs was still lower than hDPSCs that were exposed to the two hydraulic cements.

There are four main types of cell proliferation assays: i) metabolic cell proliferation assays such as those based on the reduction of tetrazolium salts, ii) DNA synthesis cell proliferation assays, iii) detection of cell proliferation markers such as the Ki-67 protein expressed during the S, G2 and M phases of the cell cycle, and iv) bioluminescence-based detection of adenosine triphosphate. In the present study, the MTT assay and quantification of DNA content were employed for examining the effects of the hydraulic cements on cell proliferation. The CyQUANT Cell Proliferation Assay Kit only quantitates, independent of cellular metabolism, the relative number of cells in a cohort based on their total DNA content, but does not recognize DNA synthesis. The latter involves incorporation of ^3^H-thymidine or a thymidine analog such as 5-bromo-2′-deoxyuridine into nascent DNA during the S phase of the cell cycle when those cells are actively proliferating^52^. Although not as precise as assays that quantitate DNA synthesis, measuring cell numbers with a DNA-binding fluorescent dye represents an indirect indicator of cell proliferation, which is a reasonable alternative for comparing the cytotoxic effects in different groups that have the same initial cell numbers.

**Figure 5:**
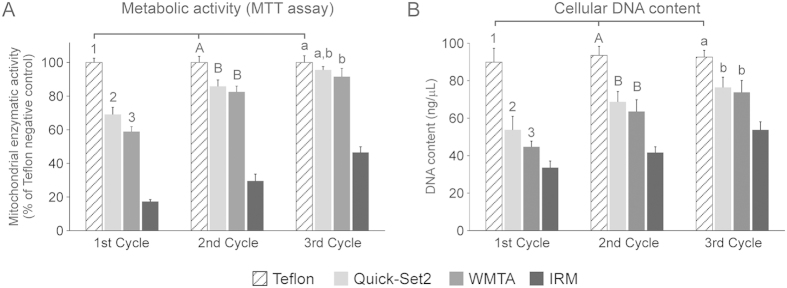
Results of cell proliferation assays after the hDPSCs were exposed to materials derived from the 3 aging cycles. (**A**) MTT assay. Mitochondrial dehydrogenase activities of hDPSCs exposed to different materials are expressed as percentages relative to the unexposed hDPSCs negative control (100%). (**B**) Cellular DNA content. Statistical analyses were only conducted for hDPSCs exposed to the two hydraulic cements and unexposed hDPSCs (negative control) from the 3 aging periods. For the factor “material” in each chart, groups labeled with the same designators (numerals for 1^st^ cycle, upper case letters for 2^nd^ cycle and lower case letters for 3^rd^ cycle) are not significantly different (P > 0.05). For the factor “aging cycle” in each chart, cycles from the same hydraulic cement that are connected with a horizontal bar are not significantly different (P > 0.05). For unexposed hDPSCs, there are no differences in activities among the 3 cycles (P > 0.05; horizontal bar not shown).

For both the MTT assay (Fig. 5A) and analysis of relative DNA content (Fig. 5B), a similar trend was observed in that both materials were initially relatively cytotoxic; the cytotoxic effects of the cements on cell metabolism and cell numbers were progressively reduced after two additional aging cycles. For either assay, the factors “material” (P < 0.001), “aging cycle” (P < 0.001) and the interaction of these factors (P < 0.001) significantly affected the respective cell proliferation parameter (mitochondrial enzymatic activity for MTT assay and DNA content for the assay on cellular DNA content). For pairwise comparison of the factor “aging cycle” in each essay (only pairwise comparisons with significant differences are described), hDPSCs that were exposed to Quick-Set2 or WMTA exhibited progressive increases in the respective cell proliferation parameter in each of the three aging cycles. For the factor “material” within the 1^st^ cycle, the respective cell proliferation parameter was higher for unexposed hDPSCs compared with cells exposed to the two hydraulic cements; expression of the respective parameter by hDPSCs exposed to Quick-Set2 was higher than WMTA. For the factor “material” within the 2^nd^ cycle, the respective cell proliferation parameter was higher in unexposed hDPSCs when compared with those exposed to the two hydraulic cements. For the factor “material” within the 3^rd^ cycle in the MTT assay (Fig. 5A), mitochondrial enzymatic activity in unexposed hDPSCs was higher than hDPSCs exposed to WMTA. For the factor “material” within the 3^rd^ cycle in the assay for DNA content (Fig. 5B), the DNA content of unexposed hDPSCs was higher than hDPSCs exposed to the two hydraulic cements.

Taken together, the results of the six assays with different endpoint measurements indicate that Quick-Set2 is comparatively less cytotoxic to undifferentiated hDPSCs than WMTA after setting for 24 h. The initial cytotoxicity of WMTA and Quick-Set2 is probably due to the high pH of the set cements, caused by diffusion of Ca(OH)_2_ into the environmental medium. The observation that Quick-Set2 is initially less cytotoxic that WMTA may be explained by the lower pH value (~10) of the calcium aluminosilicate cement, compared with the higher pH value of the tricalcium silicate cement (~12)^2^ after setting; a pH value that is closer to physiologic pH would render the culture medium less caustic to the hDPSCs. The difference in the initial cytotoxicity profiles between the two hydraulic cements warrants rejection of the null hypothesis tested in the present study.

The initial cytotoxicity exhibited by Quick-Set2 and WMTA were significantly reduced after these cements were aged in deionized water before exposing to the hDPSCs. After two cycles of aging, the cytotoxicity profiles of the two cements were essentially similar in all six assays. Such a phenomenon is a clear reflection of Paracelsus’s classic toxicology maxim on dose/response relationship: “*Alle Ding sind Gift und nichts ohn Gift; alein die Dosis macht das ein Ding kein Gift ist* (All things are poison and nothing is without poison; only the dose makes a thing not a poison)”^53^. Toxicologic risks exist only in reference to the conditions under which the cells are exposed to the set hydraulic cements. The conditions present in dissociated monolayer cell cultures are non-homeostatic because there is no mechanism for elimination of toxic substances as there would have been *in vivo*. Although dissociated monolayer cell cultures are powerful models for toxicity evaluations, these models have limitations in their ability to recapitulate *in vivo* physiologic processes and cell properties. One parallel example is the cytotoxicity of borate bioactive glass^54^ under conventional static *in vitro* culture conditions, borate bioactive glasses are toxic to cells due to the release of borate ions. However, toxicity is markedly reduced under dynamic culture conditions where there is a continuous flow of the culture medium to simulate the *in vivo* responses of a living host, such as the availability of lymphatics to remove toxic substances. Cytotoxicity is a multifactorial process that involves dynamic accumulation/removal of toxic components within the cells via active transporting mechanisms and passive diffusion, apoptosis, metabolite and ROS production, biotransformation of the toxic components by intracellular or extracellular enzymes, and interactions with the immune system. Many of these processes involve cell-cell interactions and cell-extracellular matrix interactions. These important microenvironment-driven determinants of cell behavior are often lost in dissociated two-dimensional cell culture models or even three-dimensional culture models^55^. These limitations may account for the paradox that dental materials that are considered cytotoxic in cell culture are tolerable in the *in vivo* setting. Thus, the results derived from the present study should be interpreted as risk estimates of the relative rate in which healing or tissue repair occurs in the presence of the hydraulic cements. In the future, the toxicity of these cements may be examined using tooth slice explant organ culture models to circumvent some of the limitations associated with the use of dissociated cell culture models^56^.

Because hDPSCs have the potential to differentiate into specialized cells that are capable of producing mineralized tissues, it is logical, as the extension of the present research, to examine whether the differentiation and osteogenic potentials of these stem cells are affected by the hydraulic cements. While it is almost certain that the presence of toxic components in these cements will adversely affect hDPSC differentiation and hard tissue formation, it is pertinent to highlight that there are components in dental hydraulic cements (e.g. silicate) that are potential stimulants of type I collagen synthesis and mineralization^57^. The beneficial effects of these components are initially masked by the cytotoxicity of the set cements and will not be revealed until the cements are gradually depleted of their toxic constituents. The results of the present study suggest that the experimental discoloration-resistant calcium aluminosilicate cement has to be aged for at least three cycles to reduce *in vitro* cytotoxicity before its prospective enhancement on osteogenic differentiation can be determined. Research in this direction is in order.

## Conclusions

Within the limits of using a dissociated monolayer cell culture model, it may be concluded that the experimental discoloration-resistant calcium aluminosilicate endodontic cement is initially cytotoxic to hDPSCs after setting for 24 h. At this stage, the cytotoxicity profile of the improved calcium aluminosilicate cement, as determined using assays that examined the different facets of cell viability and proliferation, is significantly more favorable than that exhibited by a bismuth-oxide containing calcium silicate cement. After two cycles of aging in deionized water, the cytotoxicity profiles of these two hydraulic cements are similar, and are much less cytotoxic compared with a zinc oxide eugenol-based restorative cement. Thus, a more favorable *in vivo* tissue response is anticipated to occur. Apart from the cell biocompatibility of the material, the effect of the hydraulic cement on the osteogenic differentiation of hDPSCs is also of great importance. Investigations of these responses are in order. The results of the present study indicate that the potential beneficial effects of the discoloration-resistant calcium aluminosilicate endodontic cement on the osteogenic differentiation of hDPSCs and their osteogenesis potential are more likely to be revealed by reducing the initial cytotoxicity expressed by the set hydraulic cement via water aging.

## Additional Information

**How to cite this article**: N. Li-na *et al.* Effects of a discoloration-resistant calcium aluminosilicate cement on the viability and proliferation of undifferentiated human dental pulp stem cells. *Sci. Rep.* **5**, 17177; doi: 10.1038/srep17177 (2015).

## References

[b1] Nowicka A. *et al.* Response of human dental pulp capped with biodentine and mineral trioxide aggregate. J. Endod. 9, 743–747 (2013). 23683272 10.1016/j.joen.2013.01.005

[b2] Camilleri J. & Pitt Ford T. R. Mineral trioxide aggregate: a review of the constituents and biological properties of the material. Int. Endod. J. 39, 747–754 (2006). 16948659 10.1111/j.1365-2591.2006.01135.x

[b3] Nielsen E. P., Herfort D. & Geiker M. R. Phase equilibria of hydrated Portland cement. Cement Concrete Res. 35, 109–115 (2005).

[b4] Han L., Kodama S. & Okiji T. Evaluation of calcium-releasing and apatite-forming abilities of fast-setting calcium silicate-based endodontic materials. Int. Endod. J. 48, 124–130 (2015). 24702182 10.1111/iej.12290

[b5] Namazikhah M. S. *et al.* The effect of pH on surface hardness and microstructure of mineral trioxide aggregate. Int. Endod. J. 41, 108–116 (2008). 18042226 10.1111/j.1365-2591.2007.01325.x

[b6] Scrivener K. L., Cabiron J. L. & Letourneux R. High-performance concretes from calcium aluminate cements. Cement. Concrete. Res. 29, 1215–1223 (1999).

[b7] Scrivener K. L. Historical and present day applications of calcium aluminate cements. In: Mangabhai R. J., Glasser F. P. (eds). *Calcium Aluminate Cements 2001.*, (IOM Communications Ltd.: London, United Kingdom, 2001), pp. 3–23.

[b8] Bradbury C., Callaway P. & Double D. D. The conversion of high alumina cement/concrete. Mater. Sci. Eng. 23, 43–53 (1976).

[b9] Ann K. Y. & Cho C. G. Corrosion resistance of calcium aluminate cement concrete exposed to a chloride environment. Materials 7, 887–898 (2014). 10.3390/ma7020887 PMC5453088 28788491

[b10] Sunnegårdh-Grönberg K., van Dijken J. W., Lindberg A. & Hörstedt P. Interfacial adaptation of a calcium aluminate cement used in class II cavities, *in vivo*. Clin. Oral. Investig. 8, 75–80 (2004). 10.1007/s00784-003-0242-3 14661081

[b11] Jefferies S. R., Pameijer C. H., Appleby D. C., Boston D. & Lööf J. A bioactive dental luting cement–its retentive properties and 3-year clinical findings. Compend. Contin. Educ. Dent. 34 **Spec No** 1, 2–9 (2013). 23577551

[b12] van Dijken J. W. & Sunnegårdh-Grönberg K. A three year follow-up of posterior Doxadent restorations. Swed. Dent. J. 29, 45–51 (2005). 16035347

[b13] Kimura M. *et al.* Study of root canal cements comprising calcium aluminate. First report on cytotoxicity. Shoni. Shikagaku. Zasshi. 29, 44–54 (1991) (in Japanese). 1784865

[b14] Pandolfelli V. C., Oliveira I. R., Jacobovitz M. & Rossetto H. L. Aluminous cement-based composition for application in endodontics and cementitious product obtained thereof. World Intellectual Property Organization Patent application registration 2009;WO/2009/067774, United States Patent Office Patent application 2011:US/2011/0281241.

[b15] Aguilar F. G., Roberti Garcia L. F. & Panzeri Pires-de-Souza F. C. Biocompatibility of new calcium aluminate cement (EndoBinder). J. Endod. 38, 367–371 (2012). 22341076 10.1016/j.joen.2011.11.002

[b16] Garcia Lda F., Huck C., Menezes de Oliveira L., de Souza P. P. & de Souza Costa C. A. Biocompatibility of new calcium aluminate cement: tissue reaction and expression of inflammatory mediators and cytokines. J. Endod. 40, 2024–2029 (2014). 25266467 10.1016/j.joen.2014.08.015

[b17] Castro-Raucci L. M. *et al.* Effects of a novel calcium aluminate cement on the early events of the progression of osteogenic cell cultures. Braz. Dental. J. 22, 99–104 (2011). 10.1590/s0103-64402011000200002 21537581

[b18] Reffitt D. M. *et al.* Orthosilicic acid stimulates collagen type 1 synthesis and osteoblastic differentiation in human osteoblast-like cells *in vitro*. Bone 32, 127–135 (2003). 12633784 10.1016/s8756-3282(02)00950-x

[b19] Washington J. T. *et al.* Biocompatibility and osteogenic potential of new generation endodontic materials established by using primary osteoblasts. J. Endod. 37, 1166–1170 (2011). 21763915 10.1016/j.joen.2011.05.011

[b20] Kohout G. D., He J., Primus C. M., Opperman L. A. & Woodmansey K. F. Comparison of Quick-Set and Mineral Trioxide Aggregate root-end fillings for the regeneration of apical tissues in dogs. J. Endod. 41, 248–252 (2015). 25459572 10.1016/j.joen.2014.10.005

[b21] Porter M. L. Bertó A., Primus C. M. & Watanabe I. Physical and chemical properties of new-generation endodontic materials. J. Endod. 36, 524–528 (2010). 20171376 10.1016/j.joen.2009.11.012

[b22] Primus C. M., Gutmann J. L., Breuer M. M. & Jefferies S. R. Methods of treatment of the dental pulp and filling root canals using water-based material. United States Patent Office Patent application 2014:US/2014/0121280.

[b23] Hutcheson C., Seale N. S., McWhorter A., Kerins C. & Wright J. Multi-surface composite vs stainless steel crown restorations after mineral trioxide aggregate pulpotomy: a randomized controlled trial. Pediatr. Dent. 34, 460–467 (2012). 23265162

[b24] Belobrov I. & Parashos P. Treatment of tooth discoloration after the use of white mineral trioxide aggregate. J. Endod. 37, 1017–1020 (2011). 21689563 10.1016/j.joen.2011.04.003

[b25] Camilleri J. Color stability of white mineral trioxide aggregate in contact with hypochlorite solution. J. Endod. 40, 436–440 (2014). 24565667 10.1016/j.joen.2013.09.040

[b26] Keskin C., Demiryurek E. O. & Ozyurek T. Color stabilities of calcium silicate-based materials in contact with different irrigation solutions. J. Endod. 41, 409–411 (2015). 25576203 10.1016/j.joen.2014.11.013

[b27] Marciano M. A. *et al.* Assessment of color stability of white mineral trioxide aggregate angelus and bismuth oxide in contact with tooth structure. J. Endod. 40, 1235–1240 (2014). 25069940 10.1016/j.joen.2014.01.044

[b28] Felman D. & Parashos P. Coronal tooth discoloration and white mineral trioxide aggregate. J. Endod. 39, 484–487 (2013). 23522541 10.1016/j.joen.2012.11.053

[b29] Berger T., Baratz A. Z. & Gutmann J. L. *In vitro* investigations into the etiology of mineral trioxide tooth staining. J. Conserv. Dent. 17, 526–530 (2014). 25506138 10.4103/0972-0707.144584 PMC4252924

[b30] Camilleri J., Cutajar A. & Mallia B. Hydration characteristics of zirconium oxide replaced Portland cement for use as a root-end filling material. Dent. Mater. 27, 845–854 (2011). 21571360 10.1016/j.dental.2011.04.011

[b31] Aguilar F. G., Garcia Lda F., Rossetto H. L., Pardini L. C. & Pires-de-Souza Fde C. Radiopacity evaluation of calcium aluminate cement containing different radiopacifying agents. J. Endod. 37, 67–71 (2011). 21146080 10.1016/j.joen.2010.10.001

[b32] Atari M. *et al.* Dental pulp of the third molar: a new source of pluripotent-like stem cells. J. Cell Sci. 125, 3343–3356 (2012). 22467856 10.1242/jcs.096537

[b33] Vishwanath V. R. *et al.* Differentiation of isolated and characterized human dental pulp stem cells and stem cells from human exfoliated deciduous teeth: An *in vitro* study. J. Conserv. Dent. 16, 423–428 (2013). 24082571 10.4103/0972-0707.117509 PMC3778624

[b34] Lei M. *et al.* Mesenchymal stem cell characteristics of dental pulp and periodontal ligament stem cells after *in vivo* transplantation. Biomaterials 35, 6332–6343 (2014). 24824581 10.1016/j.biomaterials.2014.04.071

[b35] Bryan T. E. *et al.* *In vitro* osteogenic potential of an experimental calcium silicate-based root canal sealer. J. Endod. 36, 1163–1169 (2010). 20630291 10.1016/j.joen.2010.03.034

[b36] Martin S. J. *et al.* Early redistribution of plasma membrane phosphatidylserine is a general feature of apoptosis regardless of the initiating stimulus: inhibition by overexpression of Bcl-2 and Abl. J. Exp. Med. 182, 1545–1556 (1995). 7595224 10.1084/jem.182.5.1545 PMC2192182

[b37] Decker T. & Lohmann-Matthes M. L. A quick and simple method for the quantitation of lactate dehydrogenase release in measurements of cellular cytotoxicity and tumor necrosis factor (TNF) activity. J. Immunol. Methods 115, 61–69 (1988). 3192948 10.1016/0022-1759(88)90310-9

[b38] Porter A. G. & Jänicke R. U. Emerging roles of caspase-3 in apoptosis. Cell Death Differ. 6, 99–104 (1999). 10200555 10.1038/sj.cdd.4400476

[b39] Ye Z. W., Zhang J., Townsend D. M. & Tew K. D. Oxidative stress, redox regulation and diseases of cellular differentiation. Biochim. Biophy. Acta. 1850, 1607–1621 (2015). 10.1016/j.bbagen.2014.11.010 PMC4433447 25445706

[b40] Mosmann T. Rapid colorimetric assay for cellular growth and survival: application to proliferation and cytotoxicity assays. J. Immunol. Methods 65, 55–63 (1983). 6606682 10.1016/0022-1759(83)90303-4

[b41] Jones L. J., Gray M., Yue S. T., Haugland R. P. & Singer V. L. Sensitive determination of cell number using the CyQUANT cell proliferation assay. J. Immunol. Methods 254, 85–98 (2001). 11406155 10.1016/s0022-1759(01)00404-5

[b42] Leist M. *et al.* Consensus report on the future of animal-free systemic toxicity testing. ALTEX. 31, 341–56 (2014). 25061899 10.14573/altex.1406091

[b43] U.S. National Research Council. Toxicity testing in the 21st century: a vision and a strategy. (Washington, DC: National Academy Press. 2007), pp. 1-17.

[b44] van Vliet E. Current standing and future prospects for the technologies proposed to transform toxicity testing in the 21st century. ALTEX. 28, 17–44 (2011). 21311848 10.14573/altex.2011.1.017

[b45] Chapin R. E. & Stedman D. B. Endless possibilities: stem cells and the vision for toxicology testing in the 21st century. Toxicol. Sci. 112, 17–22 (2009). 19703945 10.1093/toxsci/kfp202

[b46] Trosko J. E. & Chang C. C. Factors to consider in the use of stem cells for pharmaceutic drug development and for chemical safety assessment. Toxicology 270, 18–34 (2010). 19948204 10.1016/j.tox.2009.11.019

[b47] Huang G. T., Gronthos S. & Shi S. Mesenchymal stem cells derived from dental tissues vs. those from other sources: their biology and role in regenerative medicine. J. Dent. Res. 88, 792–806 (2009). 19767575 10.1177/0022034509340867 PMC2830488

[b48] Olson H. *et al.* Concordance of the toxicity of pharmaceuticals in humans and in animals. Regul. Toxicol. Pharmacol. 32, 56–67 (2000). 11029269 10.1006/rtph.2000.1399

[b49] Elmore S. Apoptosis: a review of programmed cell death. Toxicol. Pathol. 35, 495–516 (2007). 17562483 10.1080/01926230701320337 PMC2117903

[b50] Rahal A. *et al.* Oxidative stress, prooxidants, and antioxidants: the interplay. Biomed. Res. Int. 2014, 761264 (2014). 10.1155/2014/761264 PMC3920909 24587990

[b51] Ames B. N., Shigenaga M. K. & Hagen T. M. Oxidants, antioxidants, and the degenerative diseases of aging. Proc. Natl. Acad. Sci. USA 90, 7915–7922 (1993). 8367443 10.1073/pnas.90.17.7915 PMC47258

[b52] Zätterström U. K. *et al.* Comparison of BrdUrd and [3H]TdR incorporation to estimate cell proliferation, cell loss, and potential doubling time in tumor xenografts. Cytometry 13, 872–879 (1992). 1459003 10.1002/cyto.990130810

[b53] Rozman K. K. & Doull J. Paracelsus, Haber and Arndt. Toxicology 160, 191–196 (2001). 11246139 10.1016/s0300-483x(00)00447-9

[b54] Brown R. F. *et al.* Conversion of borate glass to hydroxyapatite and its effect on proliferation of MC3T3-E1 cells. J. Biomed. Mater. Res. 88A, 392–340 (2009). 10.1002/jbm.a.31679 18306284

[b55] Astashkina A., Mann B. & Grainger D. W. A critical evaluation of *in vitro* cell culture models for high-throughput drug screening and toxicity. Pharmacol. Ther. 134, 82–106 (2012). 22252140 10.1016/j.pharmthera.2012.01.001

[b56] Murray P. E., Lumley P. J., Ross H. F. & Smith A. J. Tooth slice organ culture for cytotoxicity assessment of dental materials. Biomaterials 21, 1711–1721 (2000). 10905412 10.1016/s0142-9612(00)00056-9

[b57] Eid A. A. *et al.* Effects of tricalcium silicate cements on osteogenic differentiation of human bone marrow-derived mesenchymal stem cells *in vitro*. Acta. Biomater. 10, 3327–3334 (2014). 24726977 10.1016/j.actbio.2014.04.006 PMC4058646

